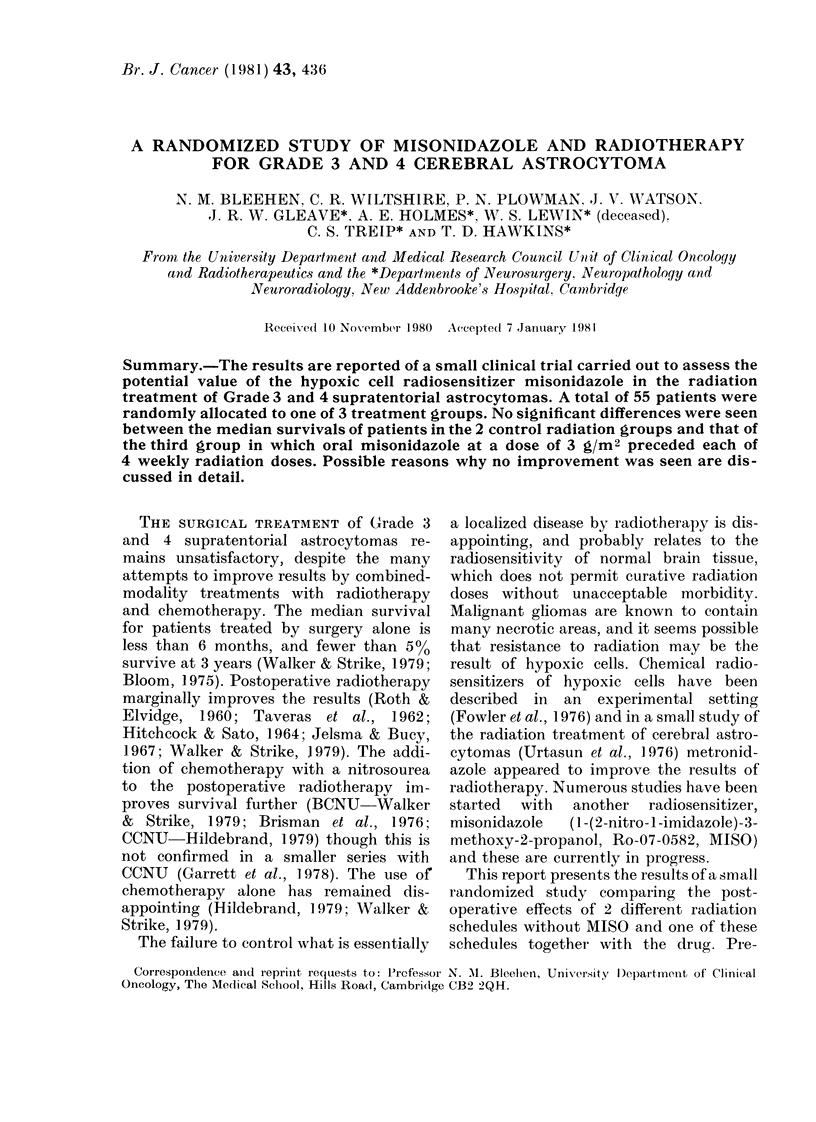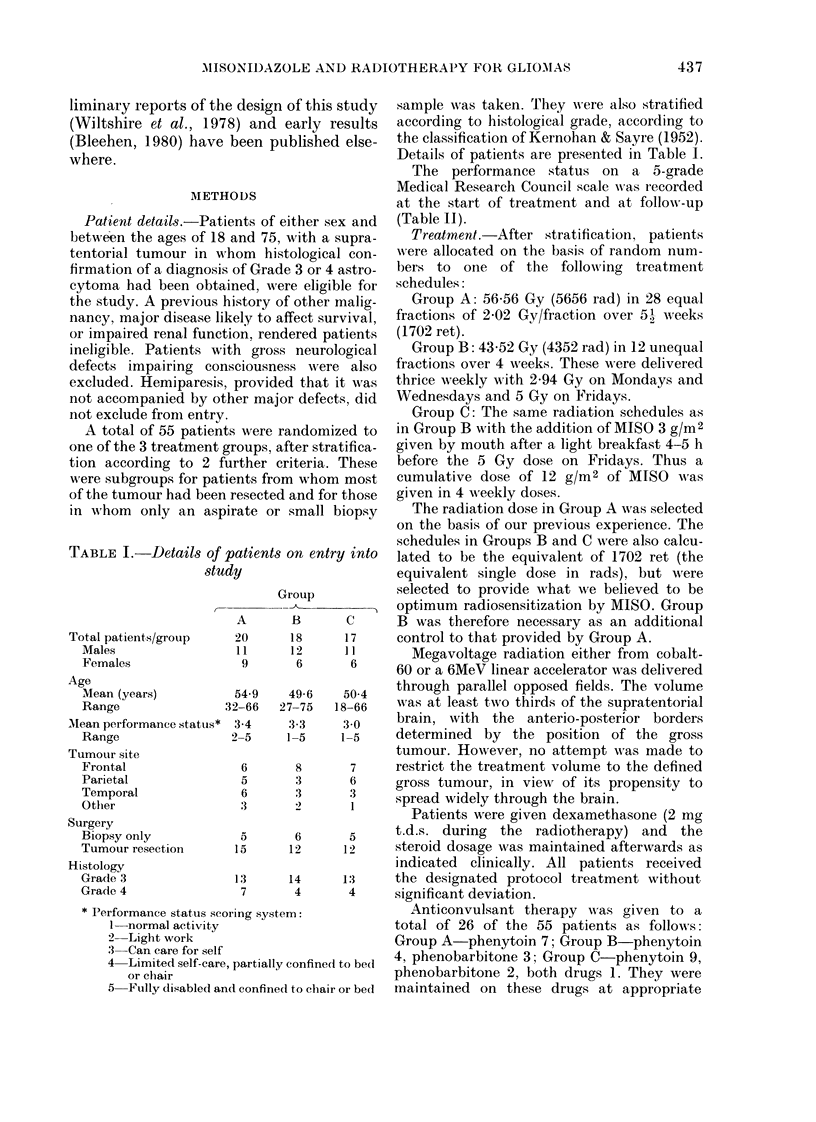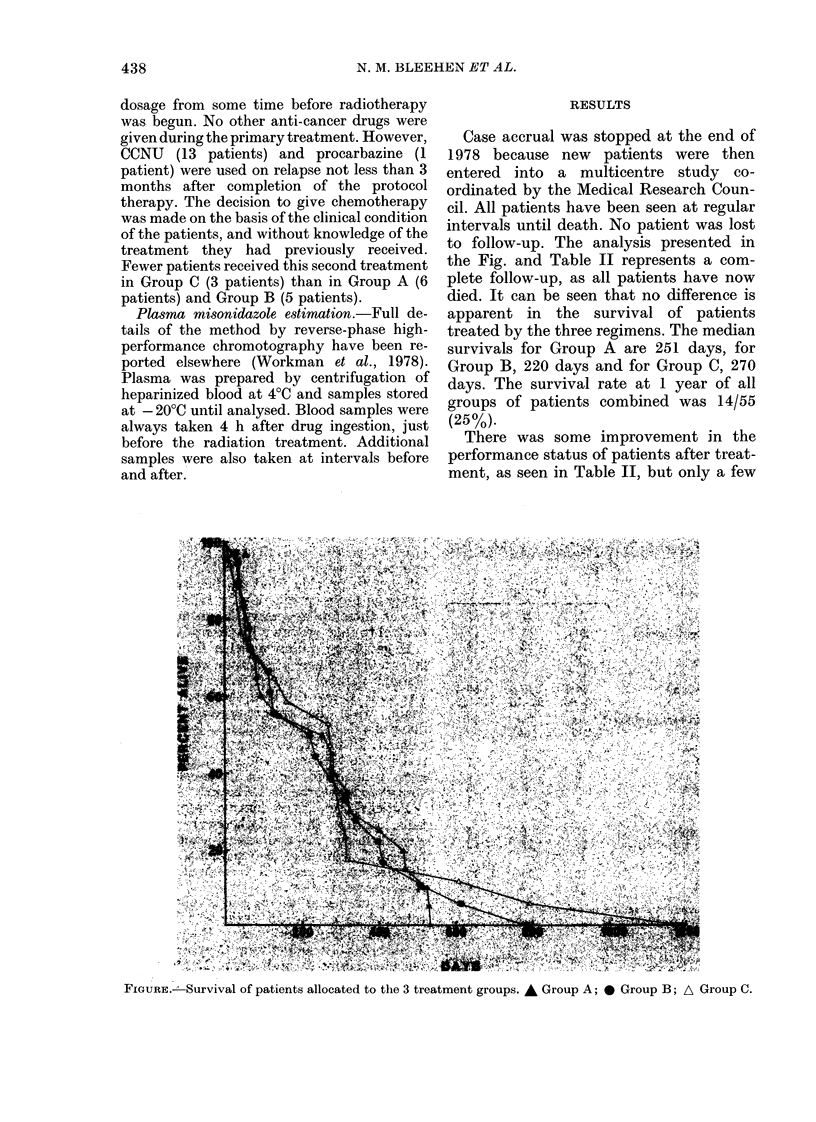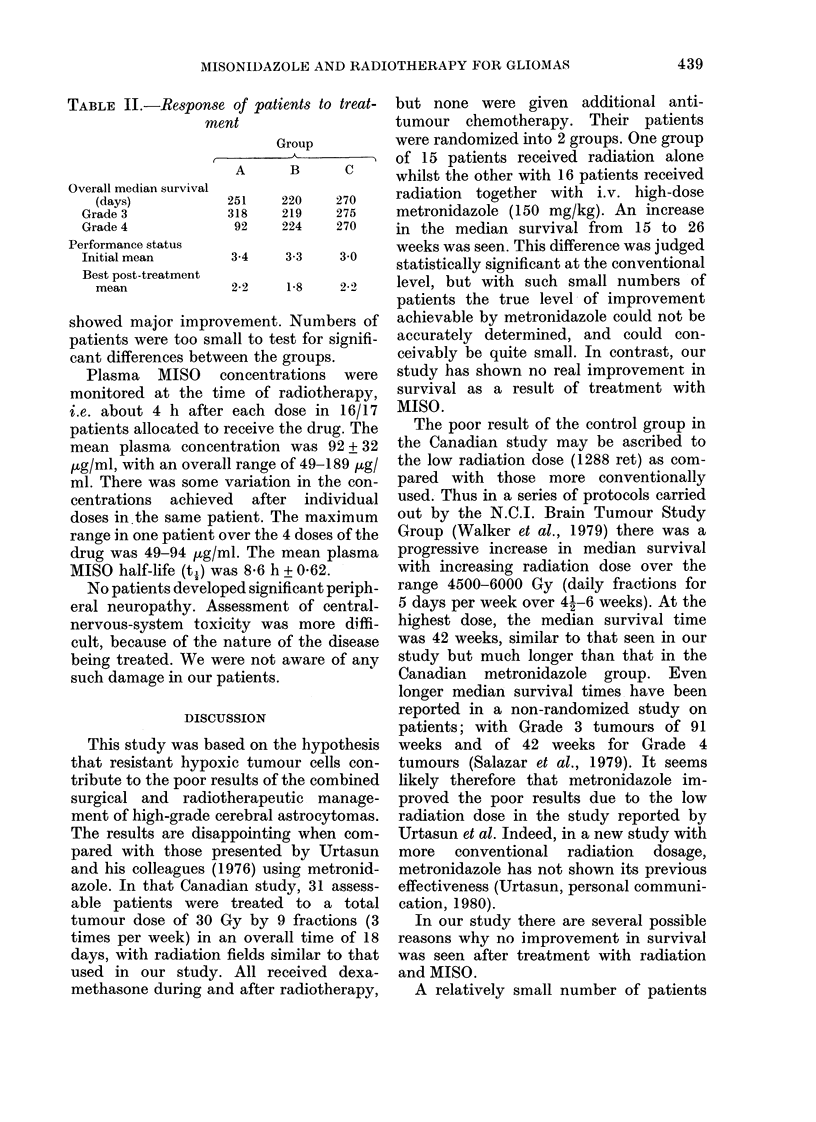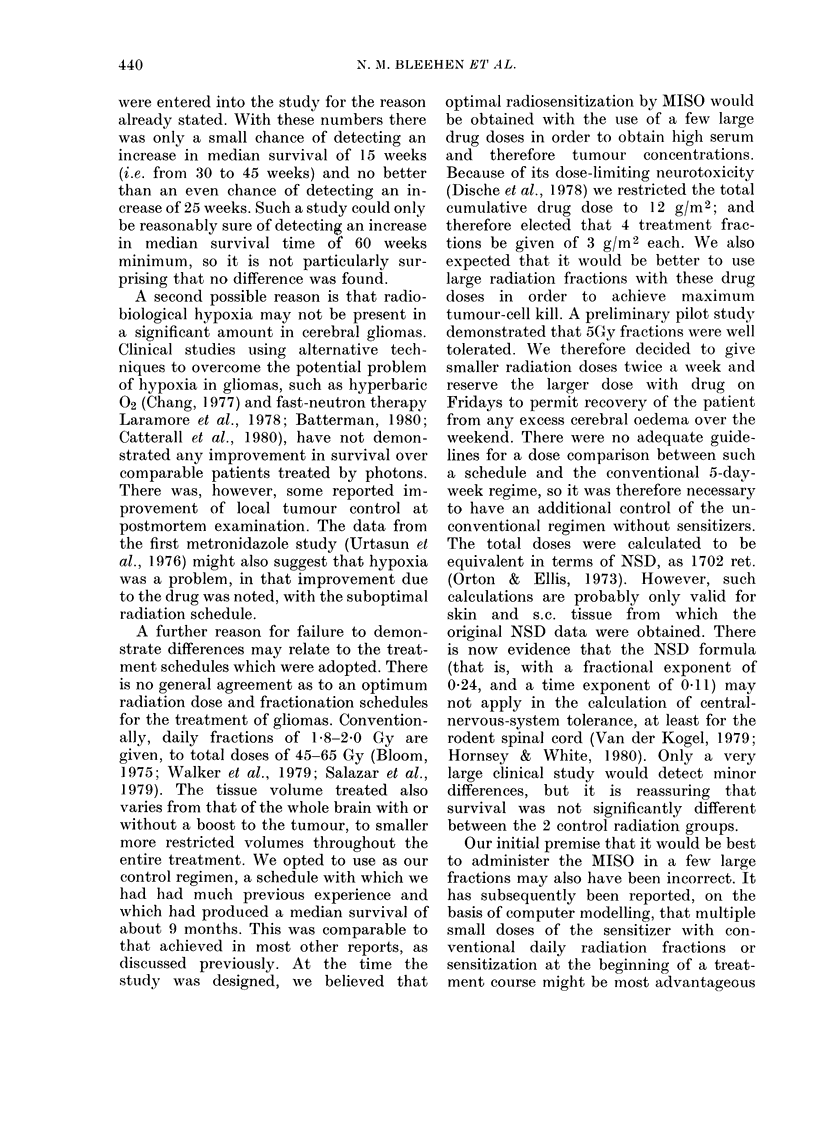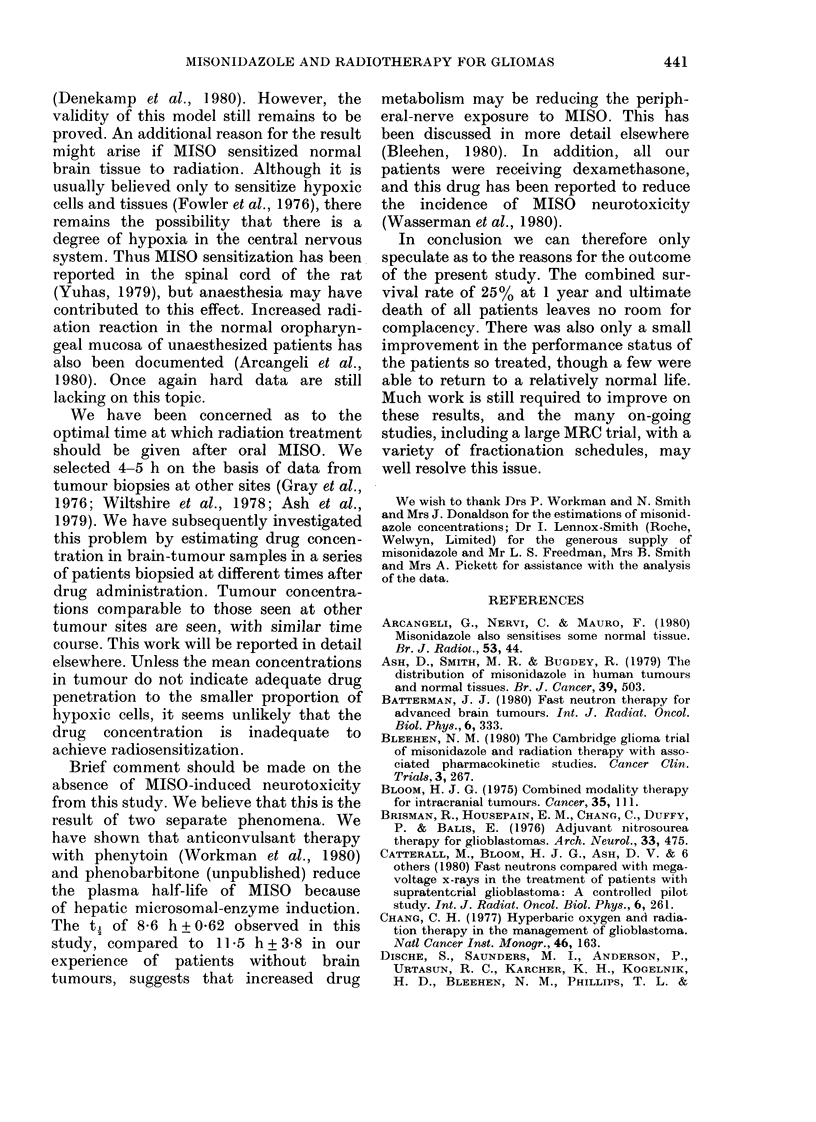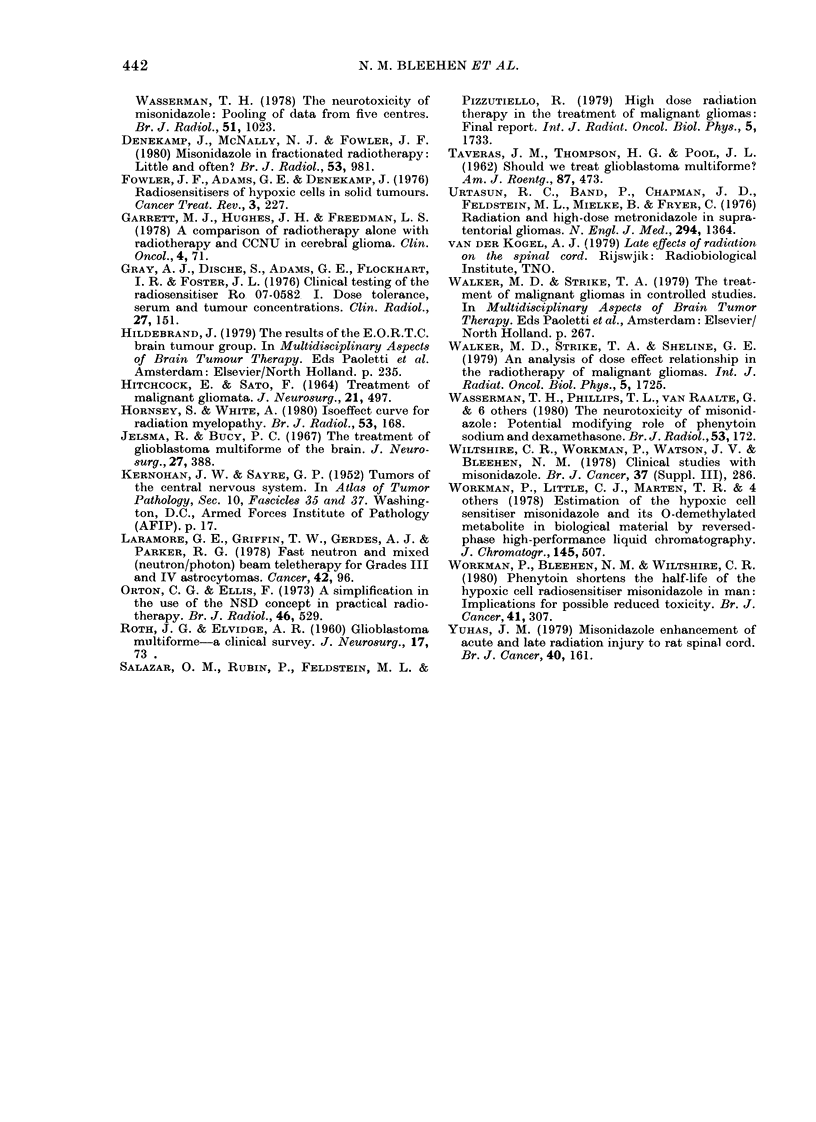# A randomized study of misonidazole and radiotherapy for grade 3 and 4 cerebral astrocytoma.

**DOI:** 10.1038/bjc.1981.64

**Published:** 1981-04

**Authors:** N. M. Bleehen, C. R. Wiltshire, P. N. Plowman, J. V. Watson, J. R. Gleave, A. E. Holmes, W. S. Lewin, C. S. Treip, T. D. Hawkins

## Abstract

The results are reported of a small clinical trial carried out to assess the potential value of the hypoxic cell radiosensitizer misonidazole in the radiation treatment of Grade 3 and 4 supratentorial astrocytomas. A total of 55 patients were randomly allocated to one of 3 treatment groups. No significant differences were seen between the median survivals of patients in the 2 control radiation groups and that of the third group in which oral misonidazole at a dose of 3 g/m2 preceded each of 4 weekly radiation doses. Possible reasons why no improvement was seen are discussed in detail.


*Br. J. Cancer* (1981) **43**, 436

### A RANDOMIZED STUDY OF MISONIDAZOLE AND RADIOTHERAPY FOR GRADE 3 AND 4 CEREBRAL ASTROCYTOMA

N. M. BLEEHEN, C. R. WILTSHIRE, P. N. PLOWMAN, J. V. WATSON, J. R. W. GLEAVE*, A. E. HOLMES*, W. S. LEWIN* (deceased), C. S. TREIP* AND T. D. HAWKINS*

*From the University Department and Medical Research Council Unit of Clinical Oncology and Radiotherapeutics and the *Departments of Neurosurgery, Neuropathology and Neuroradiology, New Addenbrooke's Hospital, Cambridge*

Received 10 November 1980 Accepted 7 January 1981

**Summary.—The results are reported of a small clinical trial carried out to assess the potential value of the hypoxic cell radiosensitizer misonidazole in the radiation treatment of Grade 3 and 4 supratentorial astrocytomas. A total of 55 patients were randomly allocated to one of 3 treatment groups. No significant differences were seen between the median survivals of patients in the 2 control radiation groups and that of the third group in which oral misonidazole at a dose of 3 g/m² preceded each of 4 weekly radiation doses. Possible reasons why no improvement was seen are discussed in detail.**

THE SURGICAL TREATMENT of Grade 3 and 4 supratentorial astrocytomas remains unsatisfactory, despite the many attempts to improve results by combined-modality treatments with radiotherapy and chemotherapy. The median survival for patients treated by surgery alone is less than 6 months, and fewer than 5% survive at 3 years (Walker & Strike, 1979; Bloom, 1975). Postoperative radiotherapy marginally improves the results (Roth & Elvidge, 1960; Taveras *et al.*, 1962; Hitchcock & Sato, 1964; Jelsma & Bucy, 1967; Walker & Strike, 1979). The addition of chemotherapy with a nitrosourea to the postoperative radiotherapy improves survival further (BCNU—Walker & Strike, 1979; Brisman *et al.*, 1976; CCNU—Hildebrand, 1979) though this is not confirmed in a smaller series with CCNU (Garrett *et al.*, 1978). The use of chemotherapy alone has remained disappointing (Hildebrand, 1979; Walker & Strike, 1979).

The failure to control what is essentially a localized disease by radiotherapy is disappointing, and probably relates to the radiosensitivity of normal brain tissue, which does not permit curative radiation doses without unacceptable morbidity. Malignant gliomas are known to contain many necrotic areas, and it seems possible that resistance to radiation may be the result of hypoxic cells. Chemical radiosensitizers of hypoxic cells have been described in an experimental setting (Fowler *et al.*, 1976) and in a small study of the radiation treatment of cerebral astrocytomas (Urtasun *et al.*, 1976) metronidazole appeared to improve the results of radiotherapy. Numerous studies have been started with another radiosensitizer, misonidazole (1-(2-nitro-1-imidazole)-3-methoxy-2-propanol, Ro-07-0582, MISO) and these are currently in progress.

This report presents the results of a small randomized study comparing the postoperative effects of 2 different radiation schedules without MISO and one of these schedules together with the drug. Pre-

Correspondence and reprint requests to: Professor N. M. Bleehen, University Department of Clinical Oncology, The Medical School, Hills Road, Cambridge CB2 2QH.

liminary reports of the design of this study (Wiltshire *et al.*, 1978) and early results (Bleehen, 1980) have been published elsewhere.

### METHODS

*Patient details.*—Patients of either sex and between the ages of 18 and 75, with a supratentorial tumour in whom histological confirmation of a diagnosis of Grade 3 or 4 astrocytoma had been obtained, were eligible for the study. A previous history of other malignancy, major disease likely to affect survival, or impaired renal function, rendered patients ineligible. Patients with gross neurological defects impairing consciousness were also excluded. Hemiparesis, provided that it was not accompanied by other major defects, did not exclude from entry.

A total of 55 patients were randomized to one of the 3 treatment groups, after stratification according to 2 further criteria. These were subgroups for patients from whom most of the tumour had been resected and for those in whom only an aspirate or small biopsy sample was taken. They were also stratified according to histological grade, according to the classification of Kernohan & Sayre (1952). Details of patients are presented in Table I.

TABLE I.—*Details of patients on entry into study*

| | Group A | Group B | Group C |
|---|---|---|---|
| Total patients/group | 20 | 18 | 17 |
| Males | 11 | 12 | 11 |
| Females | 9 | 6 | 6 |
| Age | | | |
| Mean (years) | 54·9 | 49·6 | 50·4 |
| Range | 32–66 | 27–75 | 18–66 |
| Mean performance status* | 3·4 | 3·3 | 3·0 |
| Range | 2–5 | 1–5 | 1–5 |
| Tumour site | | | |
| Frontal | 6 | 8 | 7 |
| Parietal | 5 | 3 | 6 |
| Temporal | 6 | 3 | 3 |
| Other | 3 | 2 | 1 |
| Surgery | | | |
| Biopsy only | 5 | 6 | 5 |
| Tumour resection | 15 | 12 | 12 |
| Histology | | | |
| Grade 3 | 13 | 14 | 13 |
| Grade 4 | 7 | 4 | 4 |

* Performance status scoring system:

1—normal activity

2—Light work

3—Can care for self

4—Limited self-care, partially confined to bed or chair

5—Fully disabled and confined to chair or bed

The performance status on a 5-grade Medical Research Council scale was recorded at the start of treatment and at follow-up (Table II).

*Treatment.*—After stratification, patients were allocated on the basis of random numbers to one of the following treatment schedules:

Group A: 56·56 Gy (5656 rad) in 28 equal fractions of 2·02 Gy/fraction over $5\frac{1}{2}$ weeks (1702 ret).

Group B: 43·52 Gy (4352 rad) in 12 unequal fractions over 4 weeks. These were delivered thrice weekly with 2·94 Gy on Mondays and Wednesdays and 5 Gy on Fridays.

Group C: The same radiation schedules as in Group B with the addition of MISO 3 g/m$^2$ given by mouth after a light breakfast 4–5 h before the 5 Gy dose on Fridays. Thus a cumulative dose of 12 g/m$^2$ of MISO was given in 4 weekly doses.

The radiation dose in Group A was selected on the basis of our previous experience. The schedules in Groups B and C were also calculated to be the equivalent of 1702 ret (the equivalent single dose in rads), but were selected to provide what we believed to be optimum radiosensitization by MISO. Group B was therefore necessary as an additional control to that provided by Group A.

Megavoltage radiation either from cobalt-60 or a 6MeV linear accelerator was delivered through parallel opposed fields. The volume was at least two thirds of the supratentorial brain, with the anterio-posterior borders determined by the position of the gross tumour. However, no attempt was made to restrict the treatment volume to the defined gross tumour, in view of its propensity to spread widely through the brain.

Patients were given dexamethasone (2 mg t.d.s. during the radiotherapy) and the steroid dosage was maintained afterwards as indicated clinically. All patients received the designated protocol treatment without significant deviation.

Anticonvulsant therapy was given to a total of 26 of the 55 patients as follows: Group A—phenytoin 7; Group B—phenytoin 4, phenobarbitone 3; Group C—phenytoin 9, phenobarbitone 2, both drugs 1. They were maintained on these drugs at appropriate dosage from some time before radiotherapy was begun. No other anti-cancer drugs were given during the primary treatment. However, CCNU (13 patients) and procarbazine (1 patient) were used on relapse not less than 3 months after completion of the protocol therapy. The decision to give chemotherapy was made on the basis of the clinical condition of the patients, and without knowledge of the treatment they had previously received. Fewer patients received this second treatment in Group C (3 patients) than in Group A (6 patients) and Group B (5 patients).

*Plasma misonidazole estimation.*—Full details of the method by reverse-phase high-performance chromotography have been reported elsewhere (Workman *et al.*, 1978). Plasma was prepared by centrifugation of heparinized blood at 4°C and samples stored at −20°C until analysed. Blood samples were always taken 4 h after drug ingestion, just before the radiation treatment. Additional samples were also taken at intervals before and after.

RESULTS

Case accrual was stopped at the end of 1978 because new patients were then entered into a multicentre study coordinated by the Medical Research Council. All patients have been seen at regular intervals until death. No patient was lost to follow-up. The analysis presented in the Fig. and Table II represents a complete follow-up, as all patients have now died. It can be seen that no difference is apparent in the survival of patients treated by the three regimens. The median survivals for Group A are 251 days, for Group B, 220 days and for Group C, 270 days. The survival rate at 1 year of all groups of patients combined was 14/55 (25%).

There was some improvement in the performance status of patients after treatment, as seen in Table II, but only a few

FIGURE.—Survival of patients allocated to the 3 treatment groups. ▲ Group A; ● Group B; △ Group C.

TABLE II.—*Response of patients to treatment*

| | Group | | |
|---|---|---|---|
| | A | B | C |
| Overall median survival (days) | 251 | 220 | 270 |
| Grade 3 | 318 | 219 | 275 |
| Grade 4 | 92 | 224 | 270 |
| Performance status | | | |
| Initial mean | 3·4 | 3·3 | 3·0 |
| Best post-treatment mean | 2·2 | 1·8 | 2·2 |

showed major improvement. Numbers of patients were too small to test for significant differences between the groups.

Plasma MISO concentrations were monitored at the time of radiotherapy, *i.e.* about 4 h after each dose in 16/17 patients allocated to receive the drug. The mean plasma concentration was $92 \pm 32$ μg/ml, with an overall range of 49–189 μg/ml. There was some variation in the concentrations achieved after individual doses in the same patient. The maximum range in one patient over the 4 doses of the drug was 49–94 μg/ml. The mean plasma MISO half-life ($t_{\frac{1}{2}}$) was $8{\cdot}6\ h \pm 0{\cdot}62$.

No patients developed significant peripheral neuropathy. Assessment of central-nervous-system toxicity was more difficult, because of the nature of the disease being treated. We were not aware of any such damage in our patients.

### DISCUSSION

This study was based on the hypothesis that resistant hypoxic tumour cells contribute to the poor results of the combined surgical and radiotherapeutic management of high-grade cerebral astrocytomas. The results are disappointing when compared with those presented by Urtasun and his colleagues (1976) using metronidazole. In that Canadian study, 31 assessable patients were treated to a total tumour dose of 30 Gy by 9 fractions (3 times per week) in an overall time of 18 days, with radiation fields similar to that used in our study. All received dexamethasone during and after radiotherapy, but none were given additional anti-tumour chemotherapy. Their patients were randomized into 2 groups. One group of 15 patients received radiation alone whilst the other with 16 patients received radiation together with i.v. high-dose metronidazole (150 mg/kg). An increase in the median survival from 15 to 26 weeks was seen. This difference was judged statistically significant at the conventional level, but with such small numbers of patients the true level of improvement achievable by metronidazole could not be accurately determined, and could conceivably be quite small. In contrast, our study has shown no real improvement in survival as a result of treatment with MISO.

The poor result of the control group in the Canadian study may be ascribed to the low radiation dose (1288 ret) as compared with those more conventionally used. Thus in a series of protocols carried out by the N.C.I. Brain Tumour Study Group (Walker *et al.*, 1979) there was a progressive increase in median survival with increasing radiation dose over the range 4500–6000 Gy (daily fractions for 5 days per week over 4½–6 weeks). At the highest dose, the median survival time was 42 weeks, similar to that seen in our study but much longer than that in the Canadian metronidazole group. Even longer median survival times have been reported in a non-randomized study on patients; with Grade 3 tumours of 91 weeks and of 42 weeks for Grade 4 tumours (Salazar *et al.*, 1979). It seems likely therefore that metronidazole improved the poor results due to the low radiation dose in the study reported by Urtasun *et al.* Indeed, in a new study with more conventional radiation dosage, metronidazole has not shown its previous effectiveness (Urtasun, personal communication, 1980).

In our study there are several possible reasons why no improvement in survival was seen after treatment with radiation and MISO.

A relatively small number of patients were entered into the study for the reason already stated. With these numbers there was only a small chance of detecting an increase in median survival of 15 weeks (*i.e.* from 30 to 45 weeks) and no better than an even chance of detecting an increase of 25 weeks. Such a study could only be reasonably sure of detecting an increase in median survival time of 60 weeks minimum, so it is not particularly surprising that no difference was found.

A second possible reason is that radiobiological hypoxia may not be present in a significant amount in cerebral gliomas. Clinical studies using alternative techniques to overcome the potential problem of hypoxia in gliomas, such as hyperbaric $O_2$ (Chang, 1977) and fast-neutron therapy Laramore *et al.*, 1978; Batterman, 1980; Catterall *et al.*, 1980), have not demonstrated any improvement in survival over comparable patients treated by photons. There was, however, some reported improvement of local tumour control at postmortem examination. The data from the first metronidazole study (Urtasun *et al.*, 1976) might also suggest that hypoxia was a problem, in that improvement due to the drug was noted, with the suboptimal radiation schedule.

A further reason for failure to demonstrate differences may relate to the treatment schedules which were adopted. There is no general agreement as to an optimum radiation dose and fractionation schedules for the treatment of gliomas. Conventionally, daily fractions of 1·8–2·0 Gy are given, to total doses of 45–65 Gy (Bloom, 1975; Walker *et al.*, 1979; Salazar *et al.*, 1979). The tissue volume treated also varies from that of the whole brain with or without a boost to the tumour, to smaller more restricted volumes throughout the entire treatment. We opted to use as our control regimen, a schedule with which we had had much previous experience and which had produced a median survival of about 9 months. This was comparable to that achieved in most other reports, as discussed previously. At the time the study was designed, we believed that optimal radiosensitization by MISO would be obtained with the use of a few large drug doses in order to obtain high serum and therefore tumour concentrations. Because of its dose-limiting neurotoxicity (Dische *et al.*, 1978) we restricted the total cumulative drug dose to 12 g/m$^2$; and therefore elected that 4 treatment fractions be given of 3 g/m$^2$ each. We also expected that it would be better to use large radiation fractions with these drug doses in order to achieve maximum tumour-cell kill. A preliminary pilot study demonstrated that 5Gy fractions were well tolerated. We therefore decided to give smaller radiation doses twice a week and reserve the larger dose with drug on Fridays to permit recovery of the patient from any excess cerebral oedema over the weekend. There were no adequate guidelines for a dose comparison between such a schedule and the conventional 5-day-week regime, so it was therefore necessary to have an additional control of the unconventional regimen without sensitizers. The total doses were calculated to be equivalent in terms of NSD, as 1702 ret. (Orton & Ellis, 1973). However, such calculations are probably only valid for skin and s.c. tissue from which the original NSD data were obtained. There is now evidence that the NSD formula (that is, with a fractional exponent of 0·24, and a time exponent of 0·11) may not apply in the calculation of central-nervous-system tolerance, at least for the rodent spinal cord (Van der Kogel, 1979; Hornsey & White, 1980). Only a very large clinical study would detect minor differences, but it is reassuring that survival was not significantly different between the 2 control radiation groups.

Our initial premise that it would be best to administer the MISO in a few large fractions may also have been incorrect. It has subsequently been reported, on the basis of computer modelling, that multiple small doses of the sensitizer with conventional daily radiation fractions or sensitization at the beginning of a treatment course might be most advantageous

(Denekamp *et al.*, 1980). However, the validity of this model still remains to be proved. An additional reason for the result might arise if MISO sensitized normal brain tissue to radiation. Although it is usually believed only to sensitize hypoxic cells and tissues (Fowler *et al.*, 1976), there remains the possibility that there is a degree of hypoxia in the central nervous system. Thus MISO sensitization has been reported in the spinal cord of the rat (Yuhas, 1979), but anaesthesia may have contributed to this effect. Increased radiation reaction in the normal oropharyngeal mucosa of unaesthesized patients has also been documented (Arcangeli *et al.*, 1980). Once again hard data are still lacking on this topic.

We have been concerned as to the optimal time at which radiation treatment should be given after oral MISO. We selected 4–5 h on the basis of data from tumour biopsies at other sites (Gray *et al.*, 1976; Wiltshire *et al.*, 1978; Ash *et al.*, 1979). We have subsequently investigated this problem by estimating drug concentration in brain-tumour samples in a series of patients biopsied at different times after drug administration. Tumour concentrations comparable to those seen at other tumour sites are seen, with similar time course. This work will be reported in detail elsewhere. Unless the mean concentrations in tumour do not indicate adequate drug penetration to the smaller proportion of hypoxic cells, it seems unlikely that the drug concentration is inadequate to achieve radiosensitization.

Brief comment should be made on the absence of MISO-induced neurotoxicity from this study. We believe that this is the result of two separate phenomena. We have shown that anticonvulsant therapy with phenytoin (Workman *et al.*, 1980) and phenobarbitone (unpublished) reduce the plasma half-life of MISO because of hepatic microsomal-enzyme induction. The $t_{\frac{1}{2}}$ of 8·6 h $\pm$ 0·62 observed in this study, compared to 11·5 h $\pm$ 3·8 in our experience of patients without brain tumours, suggests that increased drug metabolism may be reducing the peripheral-nerve exposure to MISO. This has been discussed in more detail elsewhere (Bleehen, 1980). In addition, all our patients were receiving dexamethasone, and this drug has been reported to reduce the incidence of MISO neurotoxicity (Wasserman *et al.*, 1980).

In conclusion we can therefore only speculate as to the reasons for the outcome of the present study. The combined survival rate of 25% at 1 year and ultimate death of all patients leaves no room for complacency. There was also only a small improvement in the performance status of the patients so treated, though a few were able to return to a relatively normal life. Much work is still required to improve on these results, and the many on-going studies, including a large MRC trial, with a variety of fractionation schedules, may well resolve this issue.

We wish to thank Drs P. Workman and N. Smith and Mrs J. Donaldson for the estimations of misonidazole concentrations; Dr I. Lennox-Smith (Roche, Welwyn, Limited) for the generous supply of misonidazole and Mr L. S. Freedman, Mrs B. Smith and Mrs A. Pickett for assistance with the analysis of the data.

### REFERENCES

ARCANGELI, G., NERVI, C. & MAURO, F. (1980) Misonidazole also sensitises some normal tissue. *Br. J. Radiol.*, **53**, 44.

ASH, D., SMITH, M. R. & BUGDEY, R. (1979) The distribution of misonidazole in human tumours and normal tissues. *Br. J. Cancer*, **39**, 503.

BATTERMAN, J. J. (1980) Fast neutron therapy for advanced brain tumours. *Int. J. Radiat. Oncol. Biol. Phys.*, **6**, 333.

BLEEHEN, N. M. (1980) The Cambridge glioma trial of misonidazole and radiation therapy with associated pharmacokinetic studies. *Cancer Clin. Trials*, **3**, 267.

BLOOM, H. J. G. (1975) Combined modality therapy for intracranial tumours. *Cancer*, **35**, 111.

BRISMAN, R., HOUSEPAIN, E. M., CHANG, C., DUFFY, P. & BALIS, E. (1976) Adjuvant nitrosourea therapy for glioblastomas. *Arch. Neurol.*, **33**, 475.

CATTERALL, M., BLOOM, H. J. G., ASH, D. V. & 6 others (1980) Fast neutrons compared with megavoltage x-rays in the treatment of patients with supratentorial glioblastoma: A controlled pilot study. *Int. J. Radiat. Oncol. Biol. Phys.*, **6**, 261.

CHANG, C. H. (1977) Hyperbaric oxygen and radiation therapy in the management of glioblastoma. *Natl Cancer Inst. Monogr.*, **46**, 163.

DISCHE, S., SAUNDERS, M. I., ANDERSON, P., URTASUN, R. C., KARCHER, K. H., KOGELNIK, H. D., BLEEHEN, N. M., PHILLIPS, T. L. &

WASSERMAN, T. H. (1978) The neurotoxicity of misonidazole: Pooling of data from five centres. *Br. J. Radiol.*, **51**, 1023.

DENEKAMP, J., MCNALLY, N. J. & FOWLER, J. F. (1980) Misonidazole in fractionated radiotherapy: Little and often? *Br. J. Radiol.*, **53**, 981.

FOWLER, J. F., ADAMS, G. E. & DENEKAMP, J. (1976) Radiosensitisers of hypoxic cells in solid tumours. *Cancer Treat. Rev.*, **3**, 227.

GARRETT, M. J., HUGHES, J. H. & FREEDMAN, L. S. (1978) A comparison of radiotherapy alone with radiotherapy and CCNU in cerebral glioma. *Clin. Oncol.*, **4**, 71.

GRAY, A. J., DISCHE, S., ADAMS, G. E., FLOCKHART, I. R. & FOSTER, J. L. (1976) Clinical testing of the radiosensitiser Ro 07-0582 I. Dose tolerance, serum and tumour concentrations. *Clin. Radiol.*, **27**, 151.

HILDEBRAND, J. (1979) The results of the E.O.R.T.C. brain tumour group. In *Multidisciplinary Aspects of Brain Tumour Therapy*. Eds Paoletti *et al.* Amsterdam: Elsevier/North Holland. p. 235.

HITCHCOCK, E. & SATO, F. (1964) Treatment of malignant gliomata. *J. Neurosurg.*, **21**, 497.

HORNSEY, S. & WHITE, A. (1980) Isoeffect curve for radiation myelopathy. *Br. J. Radiol.*, **53**, 168.

JELSMA, R. & BUCY, P. C. (1967) The treatment of glioblastoma multiforme of the brain. *J. Neurosurg.*, **27**, 388.

KERNOHAN, J. W. & SAYRE, G. P. (1952) Tumors of the central nervous system. In *Atlas of Tumor Pathology, Sec.* 10, *Fascicles 35 and 37*. Washington, D.C., Armed Forces Institute of Pathology (AFIP). p. 17.

LARAMORE, G. E., GRIFFIN, T. W., GERDES, A. J. & PARKER, R. G. (1978) Fast neutron and mixed (neutron/photon) beam teletherapy for Grades III and IV astrocytomas. *Cancer*, **42**, 96.

ORTON, C. G. & ELLIS, F. (1973) A simplification in the use of the NSD concept in practical radiotherapy. *Br. J. Radiol.*, **46**, 529.

ROTH, J. G. & ELVIDGE, A. R. (1960) Glioblastoma multiforme—a clinical survey. *J. Neurosurg.*, **17**, 73 .

SALAZAR, O. M., RUBIN, P., FELDSTEIN, M. L. & PIZZUTIELLO, R. (1979) High dose radiation therapy in the treatment of malignant gliomas: Final report. *Int. J. Radiat. Oncol. Biol. Phys.*, **5**, 1733.

TAVERAS, J. M., THOMPSON, H. G. & POOL, J. L. (1962) Should we treat glioblastoma multiforme? *Am. J. Roentg.*, **87**, 473.

URTASUN, R. C., BAND, P., CHAPMAN, J. D., FELDSTEIN, M. L., MIELKE, B. & FRYER, C. (1976) Radiation and high-dose metronidazole in supratentorial gliomas. *N. Engl. J. Med.*, **294**, 1364.

VAN DER KOGEL, A. J. (1979) *Late effects of radiation on the spinal cord*. Rijswjik: Radiobiological Institute, TNO.

WALKER, M. D. & STRIKE, T. A. (1979) The treatment of malignant gliomas in controlled studies. In *Multidisciplinary Aspects of Brain Tumor Therapy*. Eds Paoletti *et al.*, Amsterdam: Elsevier/North Holland. p. 267.

WALKER, M. D., STRIKE, T. A. & SHELINE, G. E. (1979) An analysis of dose effect relationship in the radiotherapy of malignant gliomas. *Int. J. Radiat. Oncol. Biol. Phys.*, **5**, 1725.

WASSERMAN, T. H., PHILLIPS, T. L., VAN RAALTE, G. & 6 others (1980) The neurotoxicity of misonidazole: Potential modifying role of phenytoin sodium and dexamethasone. *Br. J. Radiol.*, **53**, 172.

WILTSHIRE, C. R., WORKMAN, P., WATSON, J. V. & BLEEHEN, N. M. (1978) Clinical studies with misonidazole. *Br. J. Cancer*, **37** (Suppl. III), 286.

WORKMAN, P., LITTLE, C. J., MARTEN, T. R. & 4 others (1978) Estimation of the hypoxic cell sensitiser misonidazole and its O-demethylated metabolite in biological material by reversed-phase high-performance liquid chromatography. *J. Chromatogr.*, **145**, 507.

WORKMAN, P., BLEEHEN, N. M. & WILTSHIRE, C. R. (1980) Phenytoin shortens the half-life of the hypoxic cell radiosensitiser misonidazole in man: Implications for possible reduced toxicity. *Br. J. Cancer*, **41**, 307.

YUHAS, J. M. (1979) Misonidazole enhancement of acute and late radiation injury to rat spinal cord. *Br. J. Cancer*, **40**, 161.